# Prevalence and Predictors of Gestational Diabetes Mellitus in Sub‐Saharan Africa: A 10‐Year Systematic Review

**DOI:** 10.1002/edm2.478

**Published:** 2024-04-10

**Authors:** Daniel Ataanya Abera, Christopher Larbie, James Abugri, Mina Ofosu, Mohamed Mutocheluh, Julius Dongsogo

**Affiliations:** ^1^ Department of Laboratory Technology, Faculty of Health Sciences Kumasi Technical University Kumasi Ghana; ^2^ Department of Biochemistry and Biotechnology, Faculty of Bioscience, College of Science Kwame Nkrumah University of Science and Technology Kumasi Ghana; ^3^ Department of Biochemistry and Forensic Sciences, School of Chemical and Biochemical Sciences C. K. Tedam University of Technology and Applied Sciences Navrongo Ghana; ^4^ Department of Clinical Microbiology, School of Medical Science Kwame Nkrumah University of Science and Technology Kumasi Ghana; ^5^ Department of Biochemistry, Faculty of Biosciences University for Development Studies Tamale Ghana

**Keywords:** diabetes, Ghana, predictive factors, prevalence, sub‐Saharan Africa

## Abstract

**Background:**

Gestational diabetes mellitus (GDM) remains a global public health problem, which affects the well‐being of mothers and their children in sub‐Saharan Africa (SSA). Studies conducted in different geographical areas provide varied results on its prevalence and predictors. Understanding the extent and predictors of GDM in SSA is important for developing effective interventions and policies. Thus, this review aimed to investigate the prevalence of GDM and its predictive factors in sub‐Saharan Africa.

**Methods:**

We followed the Preferred Reporting Items for Systematic Reviews and Meta‐Analyses (PRISMA) standards in this review. An extensive search of the PubMed, Web of Sciences and EMBASE databases was carried out covering papers from 2012 to 2022 to assess the prevalence and predictors of GDM. Microsoft Excel 2019 was utilised for study management. GraphPad Prism Version 8.0 and the MedCalc statistical software were employed for data analysis. The findings were analysed using textual descriptions, tables, forest plots and heat maps.

**Results:**

Using 30 studies with 23,760 participants that satisfied the inclusion criteria, the review found the overall prevalence of GDM in SSA to be 3.05% (1.85%–4.54%). History of preterm delivery, alcohol consumption, family history of diabetes, history of stillbirths, history of macrosomia, overweight or obesity and advanced mother age were all significant predictors of gestational diabetes. Additionally, various biomarkers such as haemoglobin, adiponectin, leptin, resistin, visfatin, vitamin D, triglycerides and dietary intake type were identified as significant predictors of GDM.

**Conclusion:**

In sub‐Saharan Africa, there is a high pooled prevalence of gestational diabetes mellitus. In the light of the predictors of GDM identified in this review, it is strongly recommended to implement early screening for women at risk of developing gestational diabetes during their pregnancy. This proactive approach is essential for enhancing the overall well‐being of both mothers and children.

## Graphical abstract

Gestational diabetes mellitus (GDM) is a major health concern in sub‐Saharan Africa, impacting the well‐being of both mother and baby. A comprehensive review, following the PRISMA guidelines, investigated GDM prevalence and predictors from 2012 to 2022, identifying a 3.05% prevalence in the region. Notable predictors included preterm delivery history, alcohol consumption, family history of diabetes and various biomarkers. These findings underscore the importance of early GDM screening in sub‐Saharan Africa to enhance maternal and child health outcomes.

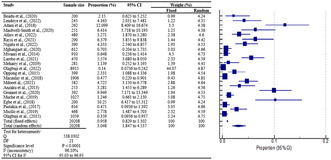

## 1 Introduction

Gestational diabetes mellitus (GDM) is still a major global health concern that has detrimental effects on the health of both the mother and the fetus [1]. GDM is the most prevalent metabolic disease during pregnancy [2], which leads to birth injuries such as shoulder dislocation, fetal metabolic disorders, bone fractures, low birthweight and nerve paralysis. GDM also brings about significant maternal and fetal problems, which includes preterm delivery, preeclampsia, premature membrane rupture, caesarean section, polyhydramnios and fetal macrosomia [3, 4, 5, 6]. GDM prevalence is estimated to be about 15% globally [7] and varies between 2% and 6% in most racial/ethnic groups studied, 10%–20% among high‐risk populations [8]. Thus, the prevalence of GDM varies by race. The prevalence of gestational diabetes in SSA is rising rapidly. The prevalence of GDM in SSA is estimated to be 14% [9]. GDM prevalence observed in a systematic review in Ethiopia reported a pooled prevalence of 12.04% [10]. In Ghana, 10% of expectant mothers had GDM as of 2014. Research carried out in Ghana between 2004 and 2015 revealed that the prevalence of GDM was 9.3% and 0.5%, respectively [11, 12]. In 2022, a study reported the prevalence of GDM in Ghana to be 8.5% [4]. Research on offspring whose mothers had prenatal diabetes has revealed that these children have an increased risk of developing type 2 diabetes, renal disease, high blood pressure, obesity, overweight and insulin resistance, and have a higher body mass index (BMI) [13, 14, 15, 16, 17]. The factors contributing to the prevalence of diabetes differ among different continents and even within individual continents [18]. Some significant risk factors of GDM include maternal obesity, previous baby with macrosomia (birthweight of more than 4.5 kg), history of gestational diabetes in previous pregnancies, family history of diabetes mellitus among first‐degree relatives and belonging to ethnic groups with high rates of diabetes mellitus [4, 19, 20].

In addition, some studies contend that genetics and other biomarkers are key factors in gestational diabetes mellitus [21]. Risk factors from the environment and genes are thought to interact to cause GDM. GDM is frequently prevalent in women who have mutations in the maturity‐onset diabetes of the young (MODY) [22]. Given the prevalence of the disease in family members, it has been hypothesised that GDM is caused by a genetic predisposition.

To avoid long‐term consequences such as cardiovascular and metabolic disorders in both the mother and the child, as well as short‐term delivery concerns, early detection of gestational diabetes mellitus is imperative. Yet, due to associated financial and logistical obstacles for populations with limited resources, GDM diagnosis in sub‐Saharan Africa is still not ideal. Targeting specialised interventions to lessen the severity and impact of the disease will be made possible by knowledge of the prevalence of diabetes and its unique risk factors in sub‐Saharan Africa. The true prevalence of GDM and its contributing variables are still unknown to policymakers, academics and service providers, although the condition continues to be a public health concern and a serious threat to Ghana's public health system as well as most other sub‐Saharan African nations. This is mostly because the majority of the information that is currently available is based on cross‐sectional data, which raises the possibility of bias and underdiagnosis. Furthermore, the majority of the investigations were carried out in various geographic regions with very disparate incidence rates and risk variables. To close this knowledge gap, the authors produced a more accurate estimate of the incidence of diabetes and the predictive risk factors associated with it by statistically synthesising data from independent research conducted in sub‐Saharan Africa.

## 2 Methodology

### 2.1 Study Protocol and Design

The Preferred Reporting Items for Systematic Reviews and Meta‐Analysis (PRISMA) criteria were followed in this review [23]. Research carried out in English in sub‐Saharan Africa between 2012 and 2022 was included in this systematic review. This review aimed to investigate the association between genetic variations, metabolic markers, micronutrients and the occurrence of gestational diabetes mellitus in SSA.

### 2.2 Types of Study Population, Exposure and Outcomes

This review examined studies that reported on the prevalence of GDM and its related factors, as well as the association between genetic variations, metabolic markers, micronutrients and GDM among pregnant women in health facilities or institutions across sub‐Saharan Africa. In cases where a study explored both diabetes mellitus and gestational diabetes mellitus, and distinguished between the two, only those that specifically investigated gestational diabetes mellitus were considered for inclusion in this review. GDM is defined as any level of glucose intolerance that arises or is first recognised during pregnancy [1]. Thus, gestational diabetes is a form of diabetes that arises during pregnancy in women who did not previously have diabetes [24].

### 2.3 Selection of Studies

The following types of articles were included in the review: (1) observational studies, including case–control, cross‐sectional and cohort studies and (2) articles that reported the prevalence of GDM, and/or associated factors or association between genetic variants, metabolic markers, micronutrients and gestational diabetes mellitus among pregnant women from respective health facilities or institutions in sub‐Saharan Africa from 2012 to 2022. The authors also looked through the reference lists of the chosen papers to find more works that fit the inclusion requirements. Articles were disqualified for at least one of the reasons listed below: (1) research not adhering to the aforementioned standards and (2) papers from conferences without abstracts, case studies or meeting reports.

### 2.4 Literature Search Strategy

The authors searched PubMed (Table S1), Web of Science (Table S2) and Embase (Table S3) databases for English‐language papers published in sub‐Saharan Africa between 2012 and 2022. The search phrases employed aligned with the corresponding databases. To find more relevant studies, the studies cited in each of the featured papers were also retrieved. The PRISMA criteria were adhered to in the analysis of the search results.

### 2.5 Protocol for Abstract/Title Screening

For the abstract/title screening procedure, there were four yes/no questions based on the inclusion criteria. The article was considered for full‐text review if all questions were answered in the affirmative (or maybe). Studies were additionally included for full‐text evaluation if the questions could not be sufficiently answered by the title or abstract alone.

1. Was the article derived from a study conducted among individuals in the sub‐Saharan African region?
2. Does the study's design align with the criteria we've set out for the desired study design?
3. Does the study, in some way, define GDM as described in the Outcomes section of our inclusion criteria?
4. Did the study evaluate the association between genetic variations, and/or metabolic markers, and/or micronutrients, and gestational diabetes mellitus, or did it determine the prevalence of GDM in a particular capacity with or without factors associated with it?

### 2.6 The Full‐Text Review Protocol

There were several yes/no questions for the whole‐article text evaluation under different headings, based on the inclusion criteria. If the answers to questions no. 1 (generic) and/or no. 2 and/or no. 3 (under particular headings) were affirmative, the article would be added for data extraction.

*General*

1. Does the study design and population satisfy all inclusion requirements as stated in the abstract/title review?

*GDM prevalence*

2 Did the publication use the data that were available to report the prevalence of GDM?

*Factors of GDM*

3 Does the study determine the prevalence of GDM in a certain capacity, with or without associated factors or does it determine the relationship, using a *p* value or 95% confidence interval, between genetic variations, metabolic markers and/or micronutrients and gestational diabetes mellitus?

### 2.7 Quality Appraisal

The JBI‐MAStARI critical assessment instrument (Appendix S1), which consists of nine questions, was employed to evaluate the articles' methodological quality. Scores were assigned to the articles according to how well they satisfied the following requirements: Absolutely, No, Not Relevant and Unclear. In addition, the Agency for Healthcare Research and Quality (AHRQ) criteria were used to evaluate the risk of bias [25].

### 2.8 Data Extraction

Endnote (version X9.2) was used to organise the studies that were searched. The studies were initially filtered based on the inclusion and exclusion criteria using the titles and abstracts. The following details were taken from each eligible study: authors, year of publication, title, study design, study location, number of study participants, sociodemographic and clinical factors, as well as micronutrients, genetics, haematological factors, *p* values and/or 95% CI. The ‘Grading of Recommendations Assessment, Development, and Evaluation’ (GRADE) method was used to examine the quality of the evidence [26].

### 2.9 Data Management

Microsoft Excel 2019 was used to extract and organise the data from the literature search. Findings were presented using GraphPad Prism, version 8.0 (GraphPad Software, San Diego, California, USA, www.graphpad.com), and the MedCalc statistical software (https://www.medcalc.org). The results were presented using texts, tables, forest plots and heat maps. The prevalence and sample size of the relevant studies were used to establish the pooled prevalence of GDM. The systematic review was reported using the PRISMA reporting guidelines.

## 3 Results

### 3.1 Eligible Reviewed Articles

**FIGURE 1:**
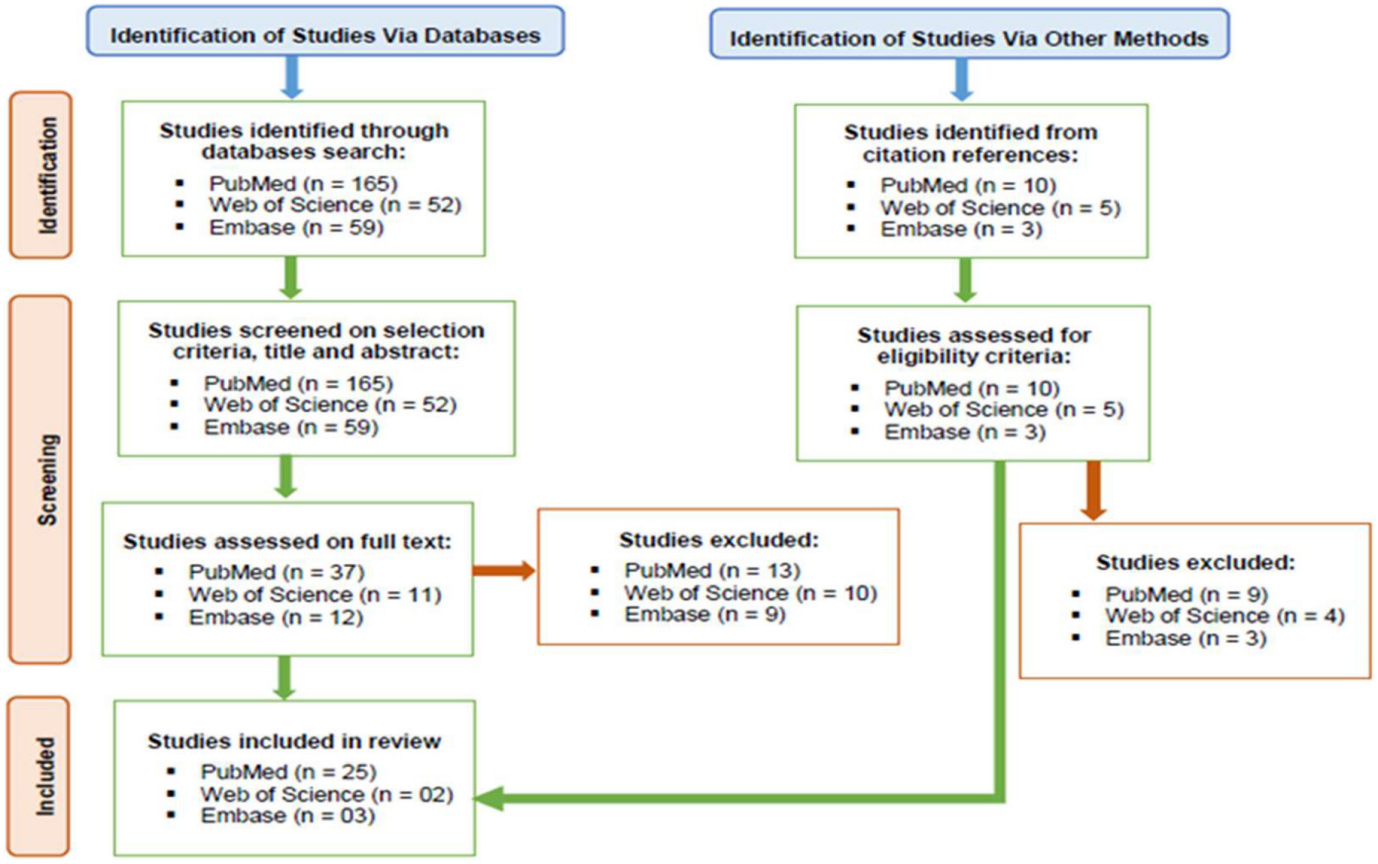
Schematic diagram of study selection in the review.

Out of the search strategies applied to the PubMed, Web of Science and Embase databases, 30 publications that satisfied all inclusion criteria were included in the review [4, 11, 27, 28, 29, 30, 31, 32, 33, 34, 35, 36, 37, 38, 39, 40, 41, 42, 43, 44, 45, 46, 47, 48, 49, 50, 51, 52, 53, 54] (Figure 1).

### 3.2 Characteristics of Studies

Of the 30 studies included in this review, two thirds (20) were cross‐sectional studies (66.67%), eight were case–control studies, and prospective cohort and prospective multicentre were one study each. Moreover, six studies were from Ethiopia, five each were from Ghana and Tanzania, respectively, four were carried out in Sudan, three were carried out in Nigeria, and two studies were from South Africa. In addition, one study each was from Cameroon, Gabon, Gambia, Kenya and Rwanda (Table 1).

**TABLE 1:** Characteristics of eligible studies included in the review.

Study	Title	Study design	Study location	Sample size	Prevalence
Boadu et al. [4]	Prevalence and Risk Factors Associated With Gestational Diabetes Mellitus Among Pregnant Women: A Cross‐Sectional Study in Ghana	Cross‐sectional	Ghana	200	4.3
Lendoye et al. [40]	Prevalence and factors associated to gestational diabetes mellitus among pregnant women in Libreville: a cross‐sectional study	Cross‐sectional	Gabon	245	10.2
Bawah et al. [32, 33]	Gestational diabetes mellitus and obstetric outcomes in a Ghanaian community	Case–control	Ghana	80/120	—
Adam et al. [27]	Association between gestational diabetes and biomarkers: a role in diagnosis	Case–control	South Africa	262	31.7
Feleke [35]	Determinants of gestational diabetes mellitus: a case–control study	Case–control	Ethiopia	567/1690	—
Maidwell‐Smith et al. [42]	Prevalence estimates of diabetes in pregnancy in a rural, sub‐Saharan population	Cross‐sectional	Gambia	251	16.1
Atlaw et al. [31]	Incidence and Factors Contributing to Gestational Diabetes Mellitus in Goba Town, Southeast Ethiopia: A Prospective Cohort Study	Cross‐sectional	Ethiopia	480	15.7
Rayis et al. [53]	High Hemoglobin Levels in Early Pregnancy and Gestational Diabetes Mellitus in Sudanese Women	Cross‐sectional	Sudan	290	18.5
Bawah et al. [32, 33]	Leptin, resistin, and visfatin as useful predictors of gestational diabetes mellitus	Case–control	Ghana	70/70	—
Nigatu et al. [49]	Prevalence of Gestational Diabetes Mellitus among pregnant women attending antenatal care clinic of St. Paul's Hospital Millennium Medical College, Addis Ababa, Ethiopia	Cross‐sectional	Ethiopia	390	16.9
Taye et al. [54]	Previous adverse pregnancy events as a predictor of gestational diabetes mellitus in Southern Ethiopia: a case–control study	Case–control	Ethiopia	80/240	—
Mghanga, Maduhu, and Nyawale [45]	Prevalence and associated factors of gestational diabetes mellitus among rural pregnant women in southern Tanzania	Cross‐sectional	Tanzania	612	4.3
Al‐Shafei et al. [28]	Maternal early pregnancy serum level of 25‐Hydroxyvitamin D and risk of gestational diabetes mellitus	Case–control	Sudan	60/60	—
Mwanri et al. [48]	Prevalence of gestational diabetes mellitus in urban and rural areas of Tanzania	Cross‐sectional	Tanzania	910	5.9
Larebo and Ermolo [39]	Prevalence and Factors Associated with Gestational Diabetes Mellitus in Women Receiving Antenatal Care at Public Hospitals in the Hadiya Zone, Southern Nation Nationality People Region	Cross‐sectional	Ethiopia	470	26.2
Meharry et al. [44]	Prevalence of gestational diabetes mellitus among women attending antenatal care at public health centers in Rwanda	Cross‐sectional	Rwanda	281	3.2
Olagbuji et al. [50]	A multicenter prospective study of early gestational diabetes mellitus: Rates, severity, and risk factors based on IADPSG‐defined fasting glycemia	Prospective multicenter	Nigeria	8915	12.5
Oppong et al. [11]	Gestational diabetes mellitus among women attending prenatal care at Korle‐Bu Teaching Hospital, Accra, Ghana	Cross‐sectional	Ghana	399	9.3
Macaulay et al. [41]	The prevalence of gestational diabetes mellitus amongst black South African women is a public health concern	Cross‐sectional	South Africa	1906	9.1
Mdoe et al. [43]	Prevalence and predictors of gestational diabetes mellitus among pregnant women attending antenatal clinic in Dodoma region, Tanzania: an analytical cross‐sectional study	Cross‐sectional	Tanzania	582	27.5
Hassan et al. [38]	Blood Groups and Hematological Parameters Do Not Associate with First Trimester Gestational Diabetes Mellitus (Institutional Experience)	Cross‐sectional	Sudan	253	
Anzaku and Musa [29]	Prevalence and associated risk factors for gestational diabetes in Jos, North‐central, Nigeria	Cross‐sectional	Nigeria	253	8.3
Grunnet et al. [36]	High Prevalence of Gestational Diabetes Mellitus in Rural Tanzania‐Diagnosis Mainly Based on Fasting Blood Glucose from Oral Glucose Tolerance Test	Prospective cohort	Tanzania	392	39
Hamdan et al. [37]	Zinc and selenium levels in women with gestational diabetes mellitus at Medani Hospital, Sudan	Case–control	Sudan	31/31	—
Muche, Olayemi, and Gete [47]	Prevalence of gestational diabetes mellitus and associated factors among women attending antenatal care at Gondar town public health facilities, Northwest Ethiopia	Cross‐sectional	Ethiopia	1027	12.8
Egbe et al. [34]	Prevalence and risk factors of gestational diabetes mellitus in a population of pregnant women attending three health facilities in Limbe, Cameroon: a cross‐sectional study	Cross‐sectional	Cameroon	200	20.5
Pastakia et al. [52]	Prevalence of gestational diabetes mellitus based on various screening strategies in western Kenya: a prospective comparison of point of care diagnostic methods	Cross‐sectional	Kenya	616	2.9
Msollo et al. [46]	Prevalence of hyperglycemia in pregnancy and influence of body fat on development of hyperglycemia in pregnancy among pregnant women in urban areas of Arusha region, Tanzania	Cross‐sectional	Tanzania	468	13
Olagbuji et al. [51]	Prevalence of and risk factors for gestational diabetes using 1999, 2013 WHO and IADPSG criteria upon implementation of a universal one‐step screening and diagnostic strategy in a sub‐Saharan African population	Cross‐sectional	Nigeria	1059	3.8
Asare‐Anane et al. [30]	Lipid Profile In Ghanaian Women With Gestational Diabetes Mellitus	Case–control	Ghana	100/100	—

### 3.3 Prevalence of Gestational Diabetes Mellitus

**FIGURE 2:**
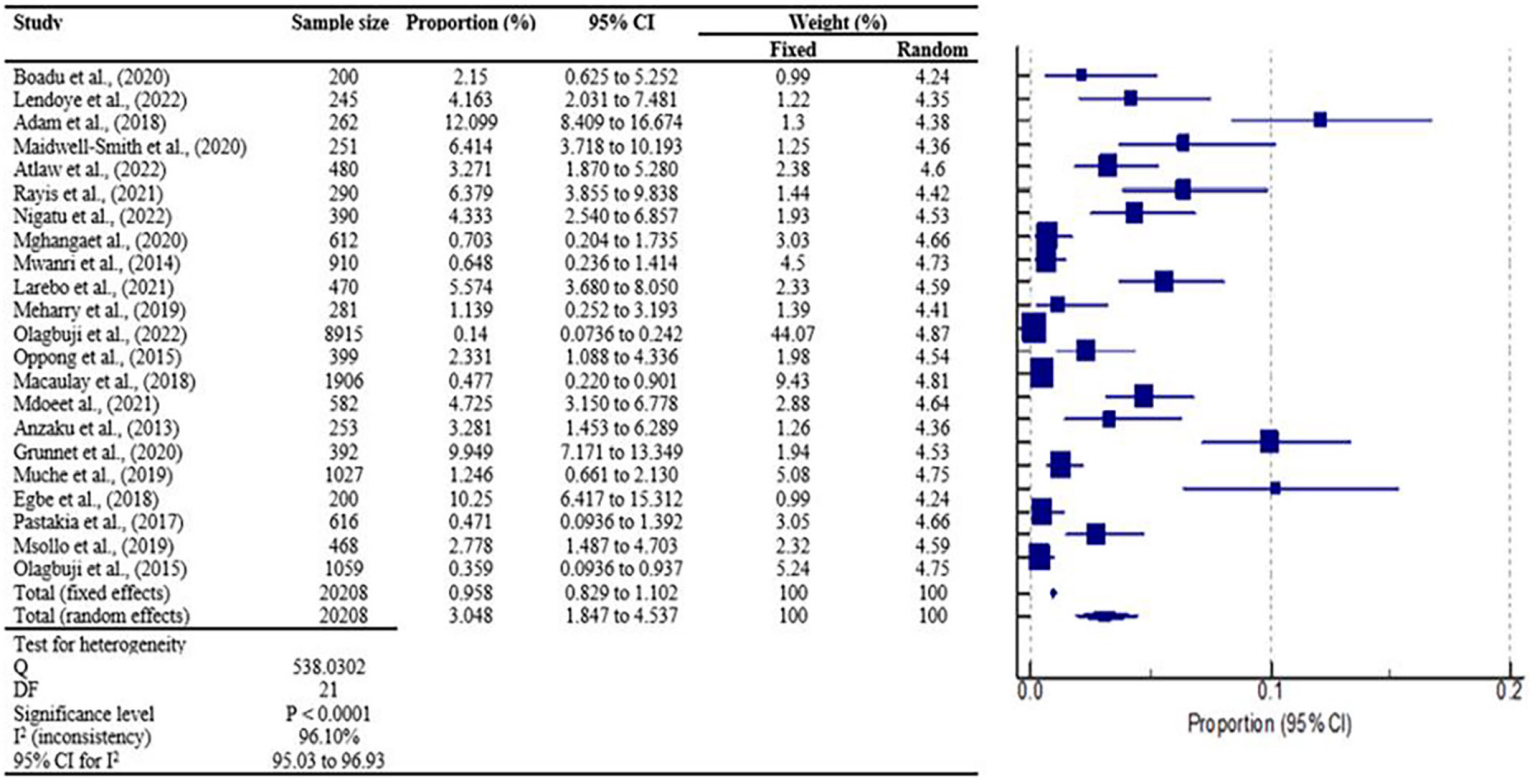
Prevalence of gestational diabetes mellitus in sub‐Saharan Africa. A forest plot representing the prevalence of gestational diabetes mellitus in sub‐Saharan Africa. The first column shows the names of the studies included, identified by the author's surname and the publication year. The second and third columns show the total study population for each respective study and the corresponding prevalence percentage (%), respectively. The fourth and fifth columns, respectively, present the estimated 95% confidence intervals for the proportions and the weights (expressed as percentages), which indicate the impact of each study on the combined result. The sixth column offers a visual depiction of the study outcomes. The small boxes positioned along the middle of the horizontal lines represent the effect estimates from individual studies, while a diamond shape represents the pooled result. The horizontal lines extending through the boxes illustrate the length of the 95% confidence interval.

A total of 22 studies were used for the pooled prevalence analysis. The MedCalc statistical program was used to calculate the 95% confidence intervals for each primary prevalence measure, which was expressed as a proportion (percentage). The cumulative effect estimate of gestational diabetes mellitus prevalence is 3.05% (1.85%–4.54%) with a 95% confidence interval and *p* < 0.0001. This results in an overall significant prevalence of gestational diabetes of approximately 3.05% in sub‐Saharan Africa, with strong heterogeneity of *I* ^2^ = 96.10% predicted to fall within the closed interval of 1.85%–4.54% (Figure 2).

### 3.4 Sociodemographic, Lifestyle and Clinical Factors Associated With GDM

**FIGURE 3:**
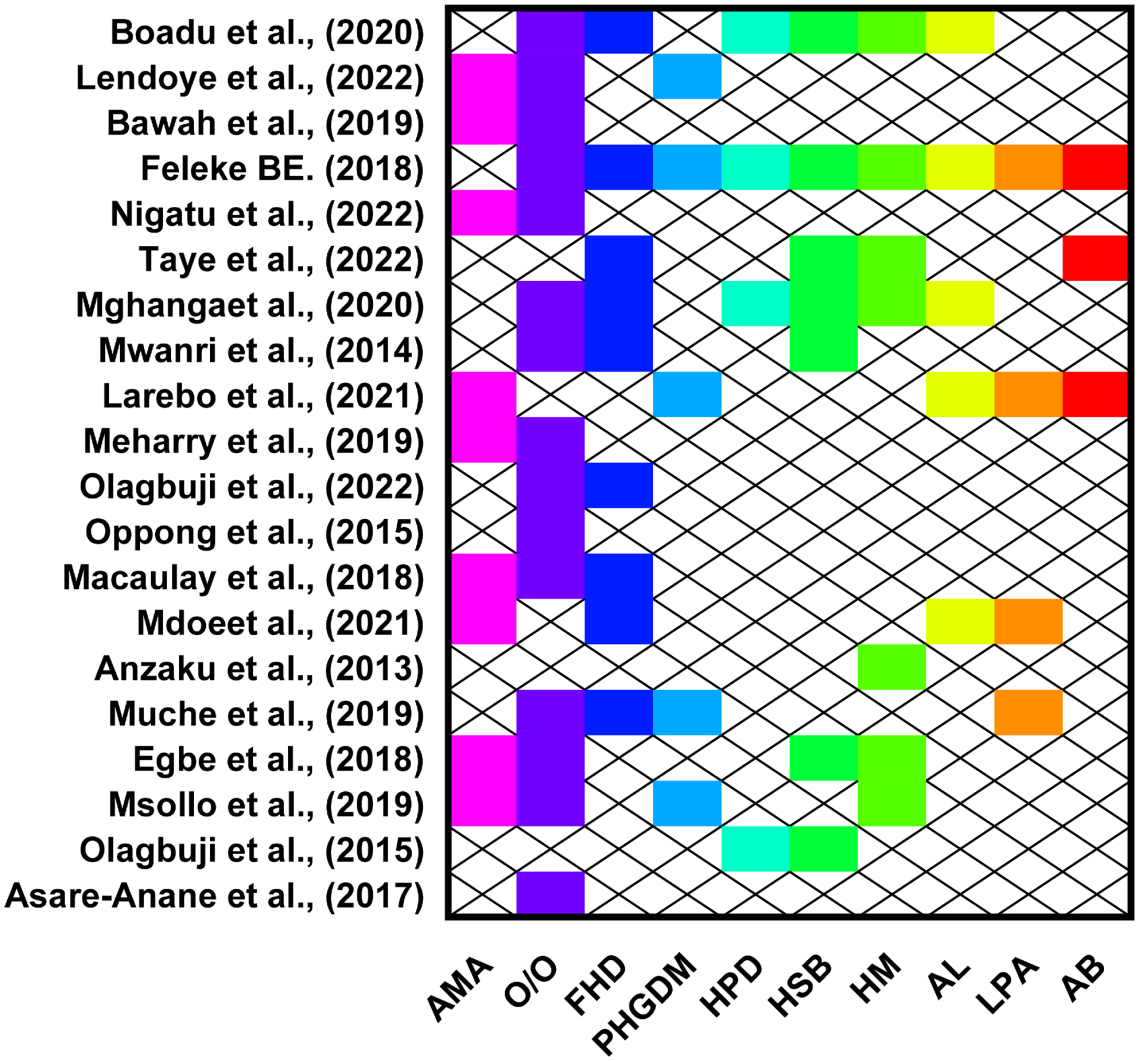
Sociodemographic and clinical factors associated with gestational diabetes mellitus. AB, abortion; AL, alcohol; AMA, advance maternal age; FHD, family history of diabetes; HM, history of macrosomia; HPD, history of preterm delivery; HSB, history of stillbirth; LPA, low physical activity; O/O, overweight or obese; PHGDM, previous history of gestational diabetes mellitus.

Of the 30 studies included in the review, 20 assessed some sociodemographic and clinical factors associated with gestational diabetes mellitus. Of the studies, 15, 9 and 9 reported overweight or obesity, advanced maternal age and family history of diabetes, respectively, were significant predictors of gestational diabetes. Moreover, seven studies each reported that having a history of stillbirth and a history of macrosomia significantly predict the chances of experiencing GDM. Having a previous history of gestational diabetes mellitus, alcohol intake and a history of preterm delivery were reported by five, five and four studies, respectively, to be significant predictors of experiencing gestational diabetes mellitus. In addition, four and three studies reported that low physical activity and abortion, respectively, significantly influence experiencing gestational diabetes (Figure 3).

### 3.5 Association Between Metabolic Markers, Micronutrients and Gestational Diabetes Mellitus

**FIGURE 4:**
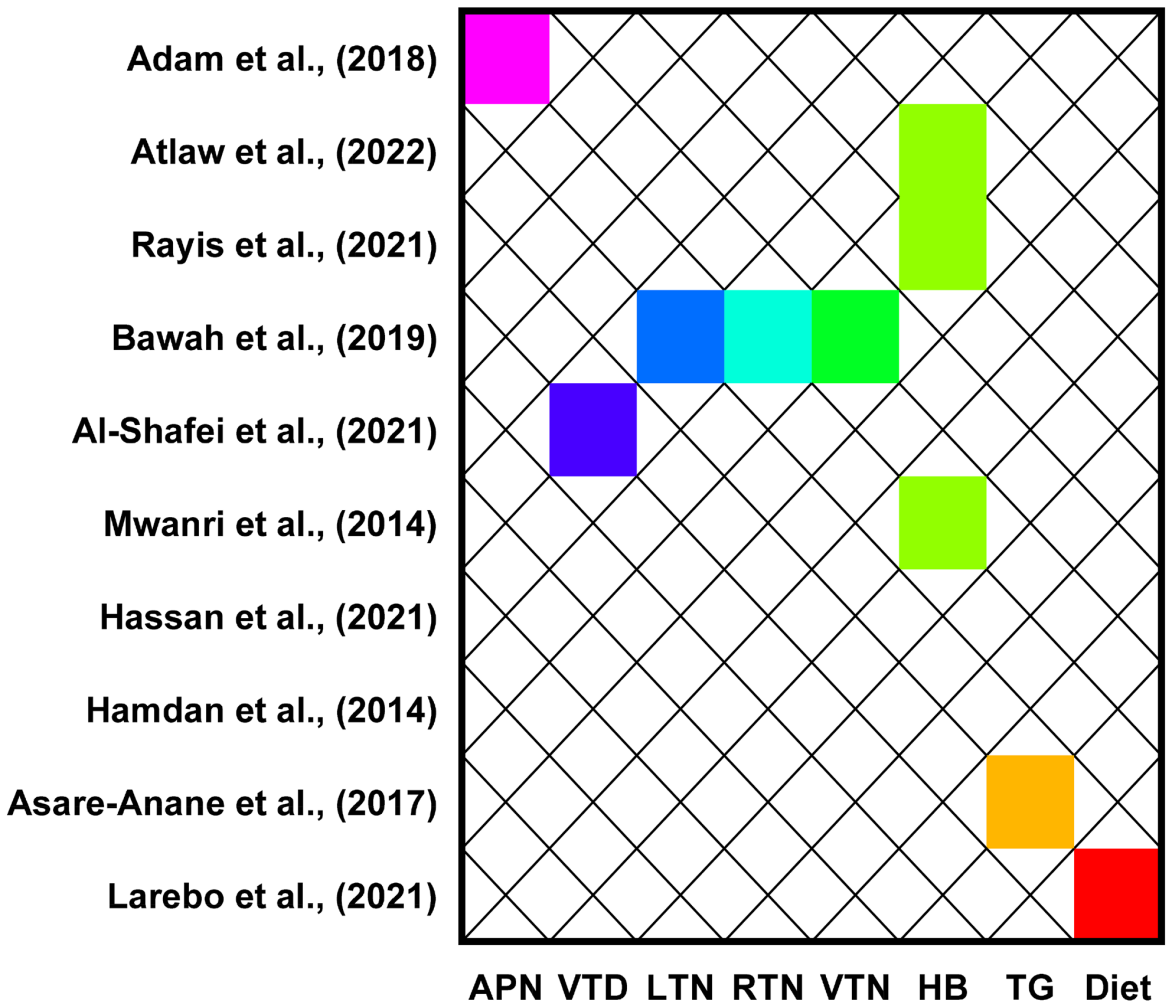
Association between metabolic markers, micronutrients and gestational diabetes mellitus. APN, adiponectin; HB, haemoglobin; LTN, leptin; RTN, resistin; TG, triglyceride; VTD, vitamin D; VTN, visfatin.

Of the studies included in the review, 10 investigated some metabolic and micronutrients associated with gestational diabetes. Of the studies, three reported that haemoglobin levels significantly predict gestational diabetes. Moreover, one study each reported adiponectin, leptin, resistin and visfatin levels were significant predictors of gestational diabetes. In addition, a study each reported that vitamin D, triglyceride and type of dietary intake significantly predict the likelihood of experiencing gestational diabetes mellitus (Figure 4).

## 4 Discussion

GDM is still a major global public health concern that poses negative consequences on both the mother's and the child's health [55]. Early detection of GDM is essential to minimise risks associated with delivery as well as long‐term consequences such as metabolic and cardiovascular disorders in both the mother and the child [56]. However, because of related financial and logistical obstacles for populations with limited resources, diagnosing GDM in sub‐Saharan Africa (SSA) is still not the best practice. Moreover, most studies conducted in different geographical locations have reported highly variable prevalence rates and risk factors. This review therefore assessed the prevalence of GDM and its predictive factors. This review found an overall prevalence of GDM to be approximately 3.05% in sub‐Saharan Africa, which is expected to fall within the closed interval of 1.85%–4.54% with substantial heterogeneity of *I* ^2^ = 96.10%. The pooled prevalence of gestational diabetes mellitus in this review aligns with previous studies conducted in various regions. Specifically, it is similar to the prevalence reported in sub‐Saharan Africa (2%–6%) by Mwanri et al. [9] and in Europe (5.4%) as per a meta‐analysis by Eades et al. [57]. However, the GDM prevalence observed in this review is notably lower than that found in a systematic review in Ethiopia conducted by Beyene et al. [10], who reported a pooled prevalence of 12.04%. It is also lower than the prevalence recorded in the United States from the National Health and Nutrition Examination Surveys between 2007 and 2014 (7.6%) [58]. Furthermore, the prevalence is lower than that reported in Asia (11.5%) [59], in eastern and south‐eastern Asia (10.1%) [60] and in Europe (10.9%) according to a systematic review by Paulo et al. [61]. The similarity of the prevalence in this study to previous studies can likely be attributed to the utilisation of similar screening methods and definitions. However, the lower prevalence of GDM observed in sub‐Saharan Africa than in Asia and Europe in previous studies may be due to variations in diagnostic methods, definitions, study populations, geographical locations and research methodologies. This study's findings further call for enhanced screening of GDM in sub‐Saharan Africa and enforcing its effective management to reduce GDM maternal and child complications.

Of the studies, overweight or obesity, advanced maternal age, family history of diabetes and having a previous history of gestational diabetes mellitus were significant predictors of gestational diabetes. These findings are consistent with Beyene et al. [10], a systematic review in Ethiopia, who reported that high body mass index, family history of diabetes and previous history of GDM were significantly associated with experiencing gestational diabetes. Similarly, a systematic review by Kiani et al. [62] in Iran found excess weight and obesity, older maternal age, family history of diabetes and history of gestational diabetes as predictors of gestational diabetes [62]. Being overweight or obese and having an abnormal metabolism are a result of inadequate dietary conditions. Obesity predisposes a person to insulin resistance and other metabolic activities. Interestingly, this current study also found dietary habits to also influence GDM. Women with advanced maternal age, and family or personal history of diabetes should be screened early and well‐monitored during pregnancy for the detection of pregnancy complications including GDM for its effective management.

Moreover, having a history of stillbirth, a history of macrosomia, a history of preterm delivery, abortion and alcohol intake were found to be significant predictors of the likelihood of experiencing gestational diabetes mellitus. This is in line with studies in Riyadh, Saudi Arabia [63], and a systemic review in Asia [59]. A systematic review by Kiani et al. [62] in Iran also found that the history of stillbirth, history of macrosomia, history of abortion and history of preterm delivery were significant predictors of gestational diabetes mellitus [62]. Uncontrolled GDM or hyperglycaemia may contribute to pregnancy difficulties such as stillbirth, macrosomia and preterm delivery; this could have a significant impact on the development of GDM in later pregnancy. Also, alcohol has detrimental effects on most metabolic pathways including glucose regulation, which may influence GDM and calls for women to avoid intake of alcohol.

In addition, low physical activity and haemoglobin levels significantly influence experiencing gestational diabetes. One crucial element of a lifestyle intervention for GDM is exercise [64]. Exercise at a moderate level for at least 30 min, four times a week, is recommended for pregnant women to be healthy. Moreover, previous studies [65, 66] show that haemoglobin levels significantly play a role in experiencing gestational diabetes among women. Clinical investigations have shown favourable benefits on serum cholesterol levels and lipid profile in addition to anaemia correction, as well as showing signs of better glucose tolerance and lowered blood pressure [67, 68].

Furthermore, this study found adiponectin, leptin, resistin and visfatin levels to be significantly associated with gestational diabetes. Also, vitamin D, triglyceride and type of dietary intake were significant predictors of experiencing gestational diabetes mellitus. This is consistent with Beyene et al. [10], a systematic review in Ethiopia which reported that inadequate dietary diversity was significantly associated with GDM [10]. A lack of dietary variety can lead to a decreased chance of acquiring a wide range of vitamins, minerals, nutrients and micronutrients essential for preventing nutrient deficiencies and chronic illnesses, thereby further predisposing women to gestational diabetes. Good dietary may also enhance markers such as adiponectin, leptin, resistin, visfatin levels and their metabolic complications [69]. These findings call for nutritional education among women especially those in the childbearing age, to reduce pregnancy complications including gestational diabetes mellitus.

This systematic review is limited by the inability to explore other relevant databases such as OVID, CINAHL or SCOPUS, which might cause the search not covering some published papers. Also, studies published in other languages other than English were excluded from the review.

## Author Contributions

**Daniel Ataanya Abera:** Data curation (equal); Formal analysis (equal); Investigation (equal); Methodology (equal); Project administration (equal); Resources (equal); Validation (equal); Writing – original draft (equal); Writing – review and editing (equal). **Christopher Larbie:** Data curation (equal); Formal analysis (equal); Methodology (equal); Supervision (equal); Writing – review and editing (equal). **James Abugri:** Investigation (equal); Methodology (equal); Supervision (equal); Writing – review and editing (equal). **Mina Ofosu:** Investigation (equal); Methodology (equal); Validation (equal); Writing – review and editing (equal). **Mohamed Mutocheluh:** Supervision (supporting); Validation (equal); Writing – review and editing (equal). **Julius Dongsogo:** Methodology (equal); Software (equal); Validation (equal); Writing – review and editing (equal).

## Conflicts of Interest

The authors declare no conflicts of interest.

## Supporting information


Table S1.
Table S2.
Table S3.

Appendix S1.

## Data Availability

This study used secondary data published on PubMed, Web of Science and EMBASE databases as no primary data were available.
