# Supplementary material for: Prevalence and Predictors of Gestational Diabetes Mellitus in Sub‐Saharan Africa: A 10‐Year Systematic Review
Source: Endocrinol Diabetes Metab. 2024 Apr 10;7(3):e00478. doi: 10.1002/edm2.478 (PMC11005715; doi:10.1002/edm2.478)
Supplement: Supplementary file 1 — Table S1. Table S2. Table S3. [file EDM2-7-e00478-s002.docx]

Table S1: PubMed Search Strategy prevalence, as well as genetic variant, metabolic markers, micro-nutrient levels in gestational diabetes mellitus in sub-Saharan Africa.

| **Search** | **Query** | **Number of hits** |
| --- | --- | --- |
| #1 | (“Gestational diabetes mellitus” OR “GDM” OR “Diabetes in pregnancy”) [All Fields) |  |
| #2 | ("Prevalence" OR "frequency" OR “burden” OR “Risk factors” OR “Factors” OR “sociodemographic factors” or “lifestyle factors” OR “clinical factors” OR “Genetic variant” OR “Metabolic markers” OR “Micronutrients” OR “Adipokines” OR “Haematological” OR “Biochemical” OR “Haematobiochemical” OR “Hematobiochemical”) [All Fields] |  |
| #3 | ("Sub-Saharan Africa" OR “Angola” OR “Burkina Faso” OR “Burundi” OR “Cape Verde” OR “Cameroon” OR “Cote d’Ivoire” OR “Central African Republic” OR “Chad” OR “Democratic Republic of Congo” OR “Congo” OR “Rwanda” OR “Comoros” OR “Eritrea” OR “Ethiopia” OR “Kenya” OR “Mauritius” OR “Somalia” OR “South Sudan” OR “Malawi” OR “Mozambique” OR “Namibia” OR “South Africa” OR “Zambia” OR “Zimbabwe” OR “Togo” OR “Benin” OR “Ivory Coast” OR “Equatorial Guinea” OR “Gabon” OR “Gambia” OR “Ghana” OR “Guinea” OR “Tanzania” OR “Uganda” OR “Botswana” OR “Eswatini” OR “Swaziland” OR “Guinea-Bissau” OR “Liberia” OR “Mali” OR “Sao Tome and Principe” OR “Mauritania” OR “Niger” OR “Nigeria” OR “Sudan” OR “Lesotho” OR “Senegal” OR “Sierra Leone” OR “Sub-Sahara” OR “Africa”) [All Fields] |  |
| #4 | (“2012/01/01" : "2022/12/31") [PDate] |  |
| #5 | #1 AND #2 AND #3 AND #4 | 165 |
|  | Eligible selected articles | 025 |

Table S2: Web of Science Search Strategy

| **Search** | **Query** | **Number of hits** |
| --- | --- | --- |
| #1 | TS= ("Prevalence" OR "frequency" OR “burden” OR “Risk factors” OR “Factors” OR “sociodemographic factors” or “lifestyle factors” OR “clinical factors” OR “Genetic variant” OR “Metabolic markers” OR “Micronutrients” OR “Adipokines” OR “Haematological” OR “Biochemical” OR “Haematobiochemical” OR “Hematobiochemical” AND “Gestational diabetes mellitus” OR “GDM” OR “Diabetes in pregnancy”) |  |
| #2 | CU= ("Sub-Saharan Africa" OR “Angola” OR “Burkina Faso” OR “Burundi” OR “Cape Verde” OR “Cameroon” OR “Cote d’Ivoire” OR “Central African Republic” OR “Chad” OR “Democratic Republic of Congo” OR “Congo” OR “Rwanda” OR “Comoros” OR “Eritrea” OR “Ethiopia” OR “Kenya” OR “Mauritius” OR “Somalia” OR “South Sudan” OR “Malawi” OR “Mozambique” OR “Namibia” OR “South Africa” OR “Zambia” OR “Zimbabwe” OR “Togo” OR “Benin” OR “Ivory Coast” OR “Equatorial Guinea” OR “Gabon” OR “Gambia” OR “Ghana” OR “Guinea” OR “Tanzania” OR “Uganda” OR “Botswana” OR “Eswatini” OR “Swaziland” OR “Guinea-Bissau” OR “Liberia” OR “Mali” OR “Sao Tome and Principe” OR “Mauritania” OR “Niger” OR “Nigeria” OR “Sudan” OR “Lesotho” OR “Senegal” OR “Sierra Leone” OR “Sub-Sahara” OR “Afr ) |  |
| #3 | Timespan= 2012-2022 |  |
| #4 | #1 AND #2 AND #3 | 52 |
|  | Eligible selected articles | 02 |

Table S3: Embase Search Strategy

| **Search** | **Query** | **Number of hits** |
| --- | --- | --- |
| #1 | ("Prevalence" OR "frequency" OR “burden” OR “Risk factors” OR “Factors” OR “sociodemographic factors” or “lifestyle factors” OR “clinical factors” OR “Genetic variant” OR “Metabolic markers” OR “Micronutrients” OR “Adipokines” OR “Haematological” OR “Biochemical” OR “Haematobiochemical” OR “Hematobiochemical” AND “Gestational diabetes mellitus” OR “GDM” OR “Diabetes in pregnancy”) |  |
| #2 | ("Sub-Saharan Africa" OR “Angola” OR “Burkina Faso” OR “Burundi” OR “Cape Verde” OR “Cameroon” OR “Cote d’Ivoire” OR “Central African Republic” OR “Chad” OR “Democratic Republic of Congo” OR “Congo” OR “Rwanda” OR “Comoros” OR “Eritrea” OR “Ethiopia” OR “Kenya” OR “Mauritius” OR “Somalia” OR “South Sudan” OR “Malawi” OR “Mozambique” OR “Namibia” OR “South Africa” OR “Zambia” OR “Zimbabwe” OR “Togo” OR “Benin” OR “Ivory Coast” OR “Equatorial Guinea” OR “Gabon” OR “Gambia” OR “Ghana” OR “Guinea” OR “Tanzania” OR “Uganda” OR “Botswana” OR “Eswatini” OR “Swaziland” OR “Guinea-Bissau” OR “Liberia” OR “Mali” OR “Sao Tome and Principe” OR “Mauritania” OR “Niger” OR “Nigeria” OR “Sudan” OR “Lesotho” OR “Senegal” OR “Sierra Leone” OR “Sub-Sahara” OR “Africa”) |  |
| #3 | Timespan= 2012-2022 |  |
| #5 | #1 AND #2 AND #3 | 59 |
|  | Eligible selected articles | 03 |
