# Supplementary figures and images for: Prevalence and Predictors of Gestational Diabetes Mellitus in Sub‐Saharan Africa: A 10‐Year Systematic Review
Source: Endocrinol Diabetes Metab. 2024 Apr 10;7(3):e00478. doi: 10.1002/edm2.478 (PMC11005715; doi:10.1002/edm2.478)

## S1 Doc- Appraisal Instruments


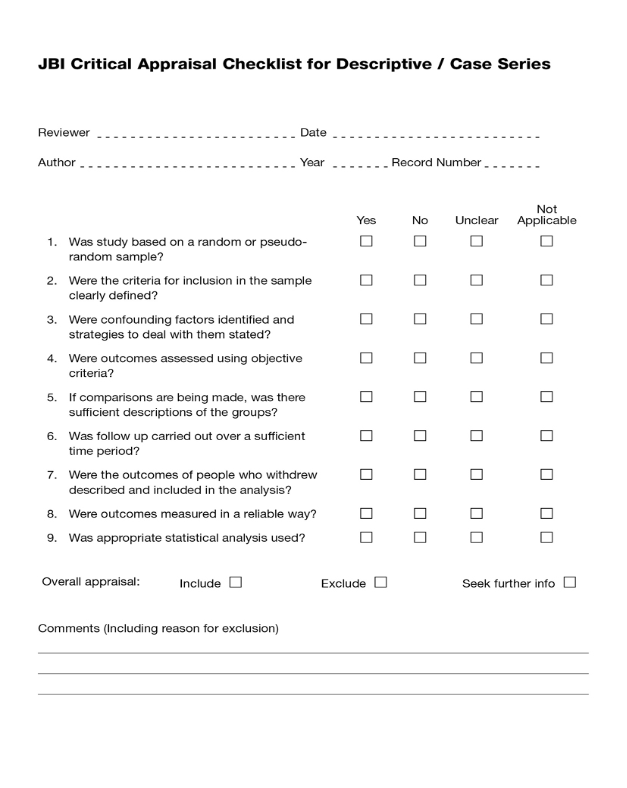

Supplement: Supplementary file 2 — Appendix S1. [file EDM2-7-e00478-s001.docx]
